# Polarity‐Engineered, Tribo‐Positive Imidazolium‐Mediated Crosslinked 6FDA‐Durene Films for Triboelectric Nanogenerator

**DOI:** 10.1002/smll.74486

**Published:** 2026-07-14

**Authors:** Sorour Faramarzi, Shabnam Yavari, Ukiliaiym Ospanova, Adilet Shynggys, Irshad Kammakakam, Haifeng Ji, Jiayi Wang, Zhen Wen, Gulnur Kalimuldina

**Affiliations:** ^1^ Department of Mechanical and Aerospace Engineering School of Engineering and Digital Sciences Nazarbayev University Astana Kazakhstan; ^2^ Department of Chemistry School of Sciences and Humanities Nazarbayev University Astana Kazakhstan; ^3^ Institute of Functional Nano and Soft Materials (FUNSOM) Joint International Research Laboratory of Carbon‐Based Functional Materials and Devices Soochow University Suzhou China

**Keywords:** crosslinked polymimides, DFT, imidazolium groups, polarity engineering, tribo‐positive

## Abstract

Expanding the library of polarity‐engineered polymers is essential for enhancing performance and designing the flexibility of triboelectric nanogenerators (TENGs). Herein, we report a polyimide (PI) film derived from an imidazolium‐mediated cross‐linked 6FDA‐durene polymer, with bromine anions as counterions, enabling surface polarity modulation and improved charge transfer. XPS and FT‐IR analyses confirm the formation of imidazolium‐mediated crosslinks in the 6FDA‐durene polyimide, verifying the successful incorporation of ionic liquid groups into the polymer structure. Density‐functional theory (DFT) calculations reveal that imidazolium crosslinking shifts the HOMO upward by 1 eV, lowering the electron‐transfer barrier and switching the triboelectric polarity from negative to positive. The fabricated TENG achieves a power density of 85.67 mW/m^2^ at 3 N and 3 Hz, lighting 81 LEDs and powering a calculator. The crosslinked material maintains stable performance over 64 800 cycles at 50°C, demonstrating thermal robustness. This polarity‐tunable, thermally stable polymer is a promising candidate for transparent TENGs in displays, windows, pavements, and hybrid energy systems.

## Graphical abstract

Imidazolium‐mediated crosslinking transforms 6FDA‐durene PI from tribo‐negative to tribo‐positive by modulating the surface electronic structure and charge‐transfer behavior. DFT calculations confirm the experimentally observed polarity transition. The polarity‐engineered polymer delivers stable triboelectric output with good thermal durability, demonstrating strong potential for operation in harsh‐climate regions and sustainable energy‐harvesting systems.

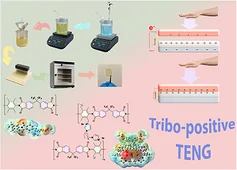

## 1 Introduction

The rapid advancement of next‐generation electronics driven by flexibility, portability, miniaturization, and the Internet of Things (IoT) has intensified the demand for battery‐independent energy sources, low‐cost, and sustainable [1]. In response, self‐powered energy‐harvesting devices have garnered significant attention for powering electronic systems and enabling autonomous sensing functions [2]. Among these, triboelectric nanogenerators (TENGs) stand out as highly promising, efficiently converting mechanical energy into electricity through triboelectrification and electrostatic induction [3]. They typically require a complementary pair of triboelectric layers: one tribo‐negative and one tribo‐positive. Significant progress has been achieved with tribo‐negative polymers possessing high electron affinity, such as Ecoflex, PTFE, and PVDF, consistently delivering enhanced output performance. In contrast, the development of tribo‐positive, electron‐donating materials has been comparatively limited. Reported candidates include covalent organic frameworks (COFs), metal oxides, and metal organic frameworks (MOFs); however, the available library remains narrow. This persistent gap highlights the critical need for discovering and engineering new tribo‐positive materials [4]. TENGs broad scalability and versatility have accelerated progress in applications ranging from wearable electronics to environmental monitoring [5]. However, the expanding scope of TENGs introduces new challenges, particularly regarding safety and reliability in environments prone to fire or ignition risks [6]. To address these concerns, two primary strategies have been proposed: (i) incorporating flame‐retardant materials to suppress or delay combustion [7], and (ii) employing thermally stable materials that maintain performance under elevated temperatures while mitigating ignition hazards. In this context, polyimide (PI) based polymers emerge as strong candidates due to their inherent thermal stability and flame resistance.

In addition, PI‐based polymers offer excellent chemical resistance and mechanical strength [8], as well as outstanding gas‐separation performance [9, 10]. These attributes have enabled its integration into a diverse array of advanced technologies, including thermal insulation, protective coatings, electronic packaging, adhesives, gas separation membranes, and liquid crystal displays. Beyond its structural and functional advantages, PI also exhibits remarkable triboelectric characteristics, serving effectively as a tribo‐negative material in TENGs [11]. This behavior is most pronounced where 4,4′‐(hexafluoroisopropylidene)diphthalic anhydride (6FDA) monomers are key moieties, a family of PI polymers, attributed to the presence of electron‐withdrawing groups such as fluorine atoms and acyl functionalities that enhance its electron affinity and facilitate efficient charge transfer during contact electrification [12]. Consequently, PI‐based TENGs have gained considerable attention for energy harvesting applications under extreme environmental conditions, including high‐temperature settings and fire detection systems [11].

Another major limitation of TENGs is their low energy conversion efficiency, largely dictated by surface charge density, which directly determines the amount of charge transferred during contact–separation. Poor charge retention further restricts their applicability in high‐power systems [13]. This parameter can be modulated by the electron affinity difference between two materials. To optimize this effect, researchers have explored physical strategies (e.g., tailoring polymer morphology and permittivity) and chemical strategies (e.g., introducing functional groups via surface modification, molecular chain grafting, or backbone functionalization) to enhance the output of TENGs [14]. Among these, molecular chain grafting and backbone functionalization are generally more advantageous than surface modification, owing to their superior structural durability. In particular, molecular chain grafting and backbone functionalization highlight the decisive role of polymer molecular structure in determining macroscopic electrification behavior. The polarity and charge trapping capacity of specific functional groups critically regulate electron transfer, thereby enabling precise modulation of the triboelectric properties. For instance, Jun Wu et al. reported that the introduction of –CF_3_ groups, owing to their strong electronegativity, enhanced the output performance of TENGs by nearly 60% in the contact‐separation mode [15]. Similarly, Jae Won Lee et al. demonstrated that sulfone and ether groups make the most significant contributions to the electrostatic potential of triboelectric materials [11]. Furthermore, Jianguo Sun et al. showed that in situ growth of zeolitic imidazolate frameworks within wood renders the material more triboelectrically positive [16]. In particular, understanding the behavior of molecular groups in amorphous polymers is critical for optimizing triboelectric materials, with density functional theory (DFT) offering valuable insights [17].

In this study, we have synthesized and investigated the experimental and theoretical properties of a polarity‐engineered imidazolium‐mediated cross‐linked 6FDA‐durene bisimidazole‐cross‐linked 6FDA‐based polymer film, which serves as a triboelectric layer for thermally stable TENG applications. A selective bromination of two sites out of four benzylic positions of pristine 6FDA‐durene PI was first conducted via a radical bromination reaction using *N*‐bromosuccinimide. A chemical crosslinking on brominated PI was further successfully performed with a newly developed bisimidazole crosslinker having a flexible –(CH_2_)_4_‐ spacer chain, yielding an imidazolium‐mediated crosslinked 6FDA‐durene film (Im‐6FDA‐durene). X‐ray photoelectron spectroscopy (XPS) and Fourier‐transform infrared spectroscopy (FT‐IR) were used for characterization; the experimental FT‐IR spectra were compared with DFT predictions to validate the computational model. Interestingly, the DFT results indicate that the incorporation of imidazolium groups via the crosslinking method converts the pristine unmodified PI material from tribo‐negative behavior to tribo‐positive behavior in the newly developed Im‐6FDA‐durene material, plausibly attributable to electron‐donating imidazolium units. The experimental results corroborate this transition: at a contact force of 3 N and frequency of 3 Hz, the device powered 81 LEDs and charged a small electronic device (a calculator), achieving a power density of 85.67 mW/m^2^. The tribo‐positive polymer also maintained stable output over 64 800 cycles at 50°C. Moreover, DFT suggests that introducing bisimidazole cross‐links modifies the electronic structure, shifting molecular‐orbital alignment, thereby favoring tribo‐positivity. Collectively, these results identify the design and development of thermal and humidity‐stable tribo‐positive polyimide materials with strong potential for hot‐humid environments triboelectric applications. Based on chemical stability and superhydrophobic properties of the crosslinked Im‐6FDA‐durene PI polymer, it can be a good candidate for future liquid‐solid triboelectric applications.

## 2 Results and Discussion

### 2.1 Characterization of the Synthesized Polymer

**FIGURE 1:**
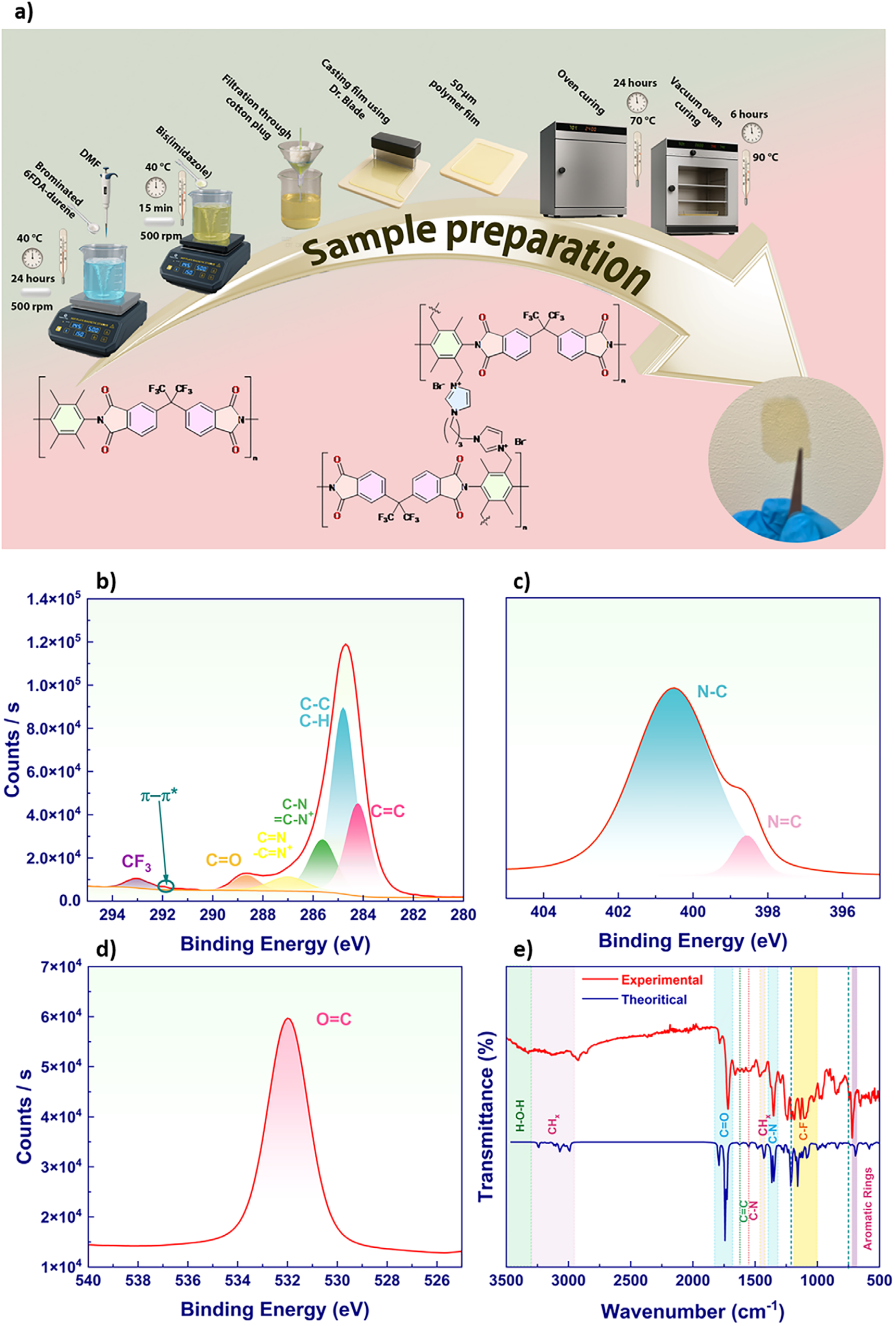
Preparation scheme and spectroscopic characterization of the imidazolium‐mediated crosslinked 6FDA‐durene polyimide. (a) Schematic illustration of the synthesis and film preparation of imidazolium‐mediated crosslinked 6FDA‐durene polymer. The corresponding chemical structures and the final transparent polymer film are also shown. XPS and FT‐IR of crosslinked Im‐6FDA‐durene PI (b) C 1s spectrum with peaks from C═C/C─C, C─H, C─N/═C─N^+^, C═N/─C═N^+^, C═O, and –CF_3_. (c) N 1s resolved into imide nitrogen (C─N) and sp^2^ nitrogen (C═N). (d) O 1s dominated by imide carbonyl (C═O). (e) Experimental and DFT FT‐IR spectra showing imide and aromatic ring modes, supporting the assigned imidazolium‐mediated crosslinked 6FDA‐durene polyimide structure.

XPS characterization was employed to investigate the surface chemical composition and bonding environments of the newly developed crosslinked Im‐6FDA‐durene PI film (Figure 1b–d). The high‐resolution C 1s spectrum was divided into several distinct peaks, corresponding to various carbon chemical states within the polymer matrix by Figure 1b. The primary peak at ∼284.1 eV is assigned to aromatic C═C bonds, originating from the phenyl rings of the polyimide backbone [18]. A secondary peak at 284.8 eV is attributed to C─C and C─H bonds, typically associated with aliphatic or non‐polar carbon environments [19]. A shoulder at 285.6 eV is assigned to C─N/═C─N^+^ in the imide and imidazolium rings, and imidazolium cations of the cross‐linked polymer [20, 21]. The C 1s component at ∼287.2 eV is attributed to C═N/─C═N^+^ bonds, consistent with nitrogen‐containing aromatic structures and imidazolium cations [21]. A higher binding energy peak at 288.7 eV is associated with imide carbonyl (C═O) bonds [22]. Furthermore, a small satellite peak in the range of ∼291.9 eV corresponds to the π–π shake‐up transition*, arising from photoelectron‐induced excitations in conjugated π‐systems, and confirms the presence of delocalized aromatic structures within both the polyimide and imidazolium components [23]. An additional broad signal at ∼293.1 eV is assigned to C─F bonds from CF_3_ groups, significantly shifted due to the strong electronegativity of fluorine atoms [24].

Moving to the N 1s spectrum (Figure 1c), two distinct peaks are observed, indicating the presence of chemically diverse nitrogen environments. The dominant peak at 400.5 eV is attributed to N─C bonds, corresponding to nitrogen atoms within imide and imidazolium rings [25, 26]. A secondary peak at ∼398.3 eV is assigned to N═C bonds, typically associated with conjugated nitrogen species in the imide functionalities. These peaks confirm both the successful incorporation of ionic nitrogen moieties and the preservation of the polyimide framework. The O 1s spectrum (Figure 1d) displays a strong, symmetric peak centered at ∼532.1 eV, which is characteristic of carbonyl oxygen (C═O) atoms in imide groups [27]. The absence of significant shoulder features suggests a single dominant oxygen environment, consistent with the designed polymer structure. This observation supports the chemical composition of crosslinked Im‐6FDA‐durene PI, which lacks oxygen‐containing functionalities such as ether (C─O─C), hydroxyl (O─H), or ester (─COOR) groups. The results collectively affirm the targeted synthesis of an imidazolium‐functionalized ionic polyimide with minimal structural defects or unintended oxygen species.

In addition, theoretical and experimental analyses using FT‐IR spectroscopy were conducted to investigate the chemical structure and confirm the functional groups present in the synthesized material (Figure 1e). The FT‐IR spectrum in the 619–770 cm^−^^1^ region provides insights into the presence and interactions of heterocyclic and aromatic units. A weak absorption near 619 cm^−^^1^ is attributed to the out‐of‐plane bending of imidazole rings. A distinct vibrational feature within the 680–725 cm^−^^1^ region is attributed to the out‐of‐plane bending of C─H bonds on substituted benzene rings, serving as a diagnostic indicator of aromatic structural motifs present within the polymer matrix [28]. The absorption at ∼725 cm^−^^1^ is consistent with imide ring deformation, affirming successful imidization [29]. A distinct peak at 750 cm^−^^1^ (dashed line) is assigned to the imidazolium cation, confirming the incorporation of ionic liquid functionalities [30]. In the 1000–1190 cm^−^^1^ region, strong bands at 1184 and 1158 cm^−^^1^ arise mainly from C─F stretching of the –CF_3_ groups in the hexafluoroisopropylidene unit (–C(CF_3_)_2_–) [31, 32]. These fluorinated groups are known to produce sharp peaks due to their high electronegativity and strong bond polarity. Additionally, a peak near 1150 cm^−^^1^ results from C─N heterocycle rings. Also, the absorption region between 1317 and 1397 cm^−^^1^ is characteristic of C─N stretching vibrations, commonly observed in polyimide structures and nitrogen‐containing heterocycles [33, 34]. More specifically, the FT‐IR bands around 1350–1360 cm^−^^1^ are frequently attributed to C─N stretching or ring deformation modes, particularly in imidazolium [35]. These bands offer strong evidence of the successful integration of imidazolium ionic functionalities within the polymer backbone, via the crosslinking method.

A prominent peak at 1209 and 1622 cm^−^^1^ is assigned to C═C stretching in the bisimidazole aromatic rings, indicating the presence of delocalized π‐electron systems [36, 37]. Also, the strong absorption bands observed between 1680 and 1825 cm^−^^1^ correspond to the asymmetric and symmetric stretching vibrations of the carbonyl (C═O) groups in imide rings [38]. This spectral feature is a hallmark of fully imidized polyimides and confirms the completion of the imidization reaction. Moreover, the broad region spanning 2950–3300 cm^−^^1^ is assigned to aromatic C─H stretching modes [39]. Importantly, no absorption bands were observed above 3300 cm^−^^1^, particularly those associated with N─H or O─H stretching vibrations. The absence of these peaks suggests the complete conversion of amine and anhydride precursors, further supporting the successful imidization process. Additionally, the lack of O─H stretching (typically between 3200–3600 cm^−^^1^) confirms that water is not retained in the polymer matrix [40]. This behavior is attributed to the hydrophobic nature of the trifluoromethyl (–CF_3_) groups, which effectively prevent moisture absorption. Additionally, thermal behavior and material stability of imidazolium‐mediated crosslinked 6FDA‐Durene PI were investigated by thermogravimetric analysis (TGA) and compared to that of the pristine 6FDA‐Durene PI, as depicted in Figure S1. While pristine PI exhibited a single degradation peak corresponding to the polyimide backbone, imidazolium‐mediated crosslinked PI displayed two‐step degradation graphs [41]. Precisely, a distinct initial weight loss in the range of 260–400°C was attributed to the imidazolium crosslinked moieties, whereas the significant polymer backbone degradation started at ca. 480°C. Overall, the TGA result further proved that imidazolium‐mediated crosslinked PI has thermal stability at elevated temperature up to 260°C, which is far beyond the minimum required for TENG applications.

**FIGURE 2:**
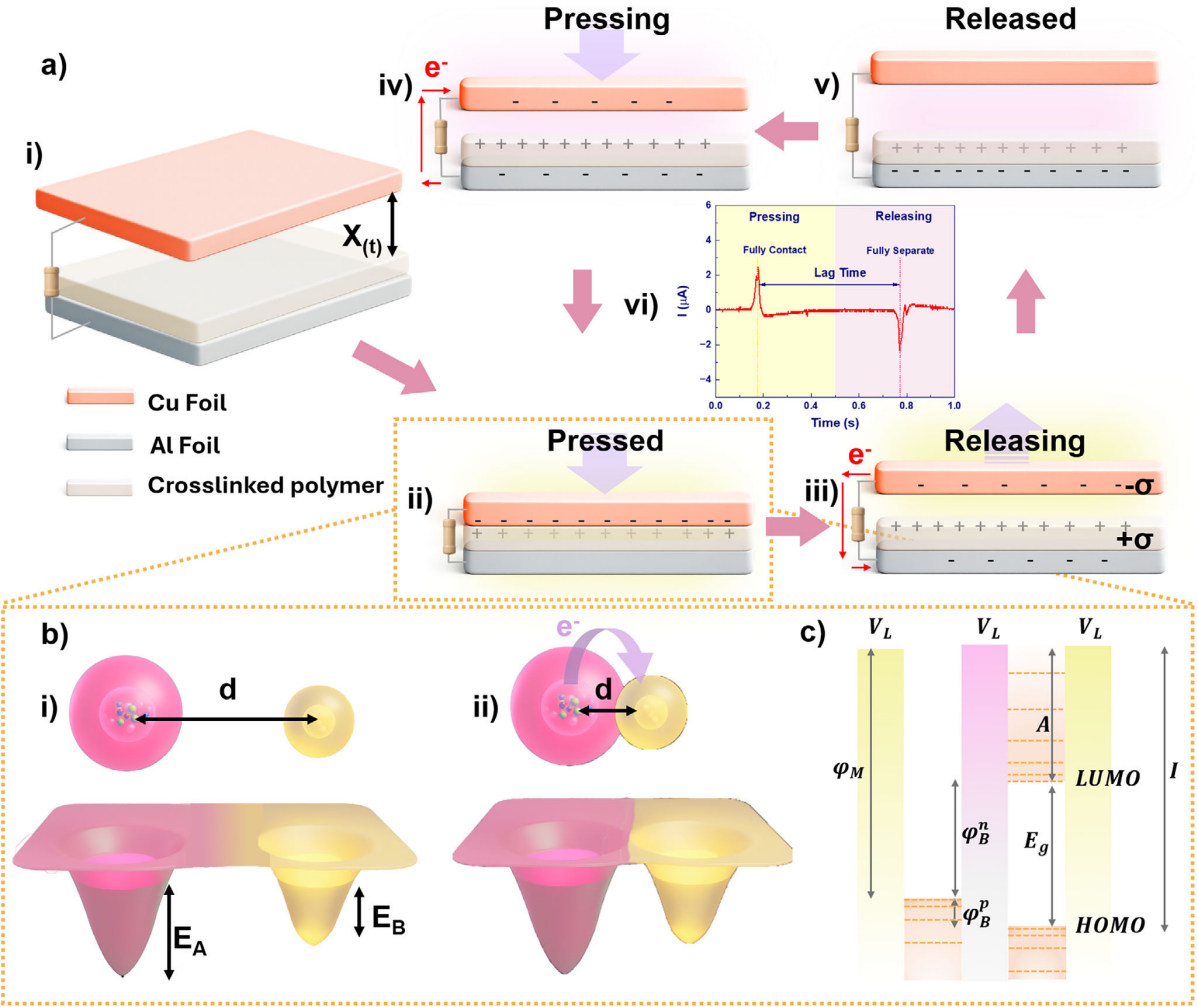
The TENG (a) working principle of newly developed crosslinked Im‐6FDA‐durene PI, (b) the electron‐cloud model during the contact electrification process and the associated charge transfer mechanism, and (c) the electronic band structure at the metal‐polymer interface.

For further clarification regarding TENG, we begin by detailing the working principle of our sample. Generally, a contact‐separation TENG consists of three layers: two electrodes and a dielectric layer. The cross‐linked 6FDA polymer, selected as the tribo‐positive layer, is attached to Al foil, while Cu foil serves as the tribo‐negative layer. Figure 2a illustrates the operating mechanism of the electron current in the contact‐separation 6FDA‐TENG, induced by external mechanical force. Initially, when the two surfaces are separated, no potential difference exists, as shown in Figure 2a‐i. Upon contact, charge transfer occurs according to the *electron‐cloud potential well model* (Figure 2b), where the electron clouds of two atoms overlap. This overlap results in negative charges on the Cu foil and positive charges on the cross‐linked polymer surface (Figure 2a‐ii). Subsequent separation drives electron flow between the electrodes through the external circuit until electrostatic equilibrium is reached (Figure 2a‐iii), at which point the transfer charge stops and the current decreases to zero (Figure 2a‐iv). Recompression of the layers induces reverse charge transfer, completing the cycle (Figure 2a‐v). These dynamic processes correspond to positive and negative current flow, as shown in Figure 2a‐vi [42]. Under open‐circuit conditions, no net current flows between the electrodes; however, a measurable open‐circuit voltage (*V_oc_*) is established due to the accumulated surface charges. Conversely, when a short circuit is applied, a transient current (*I_sc_*) is observed, driven by the potential difference. The key output parameters of the TENG, including *V_oc_*, *I_sc_*, and surface charge density (σ), can be described by the following equations:

(1)

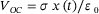

(2) $$I_{SC}=S\sigma d_{0}v\left(t\right)\;/{\left(d+x\left(t\right)\right)}^{2}$$

(3)

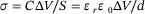



(4)

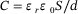




Here, σ is the surface charge density, *x*(*t*) is the time‐dependent separation distance, *v*(*t*) is the separation velocity, ε _0_ is the air permittivity, ε _r_ is the relative dielectric coefficient, *S* is the contact area, *d* is the dielectric thickness, and ΔV is the potential difference across the dielectric layer. These equations provide a theoretical framework for evaluating the TENG's electrical output and the underlying charge transfer mechanisms.

Additionally, Figure 2b provides a more detailed representation of the electron‐cloud potential well model and the associated barrier height, as discussed in the previous step (Figure 2a‐i). Initially, Figure 2b‐i illustrate the atomic clouds of two atoms when their separation exceeds the bond length. Further, when two macroscopic surfaces come into intimate contact, their electron clouds overlap, reducing the interfacial potential barrier (Figure 2b‐ii) [43]. In the contact phase, the electron acceptor provides vacant orbitals, while the electron donor supplies outer‐shell electrons, facilitating charge transfer until a new electrostatic equilibrium is reached. In this study, the transferred electrons remain on the Cu foil due to the re‐established energy barrier, resulting in a net negative charge on the Cu foil after contact electrification. The energy required to excite electrons from lower to higher orbital states is partially reduced by the barrier lowering due to electron‐cloud overlap. The remaining energy is supplied by the repulsive force that arises when interatomic distances fall below the equilibrium bond length [44], with both contributions ultimately derived from atomic kinetic energy. Moving to the barrier height at the metal‐polymer interface, the electronic structure of the two interacting solids is depicted in Figure 2c, as reported by Hisao Ishii et al. [45]. The carrier injection barrier heights for electrons ($\varphi_{B}^{n}$), and holes ($\varphi_{B}^{p}$) are represented as following equations:

(5) $$\varphi_{B}^{p}=I-\varphi_{m}$$

(6) $$\varphi_{B}^{n}=E_{g}\;-\varphi_{B}^{p}$$

where V_L_, A, I, φ_*M*_, and E_g_ denote the vacuum level, electron affinity, ionization energy, metal work function, and band gap of the polymer chain, respectively.

**FIGURE 3:**
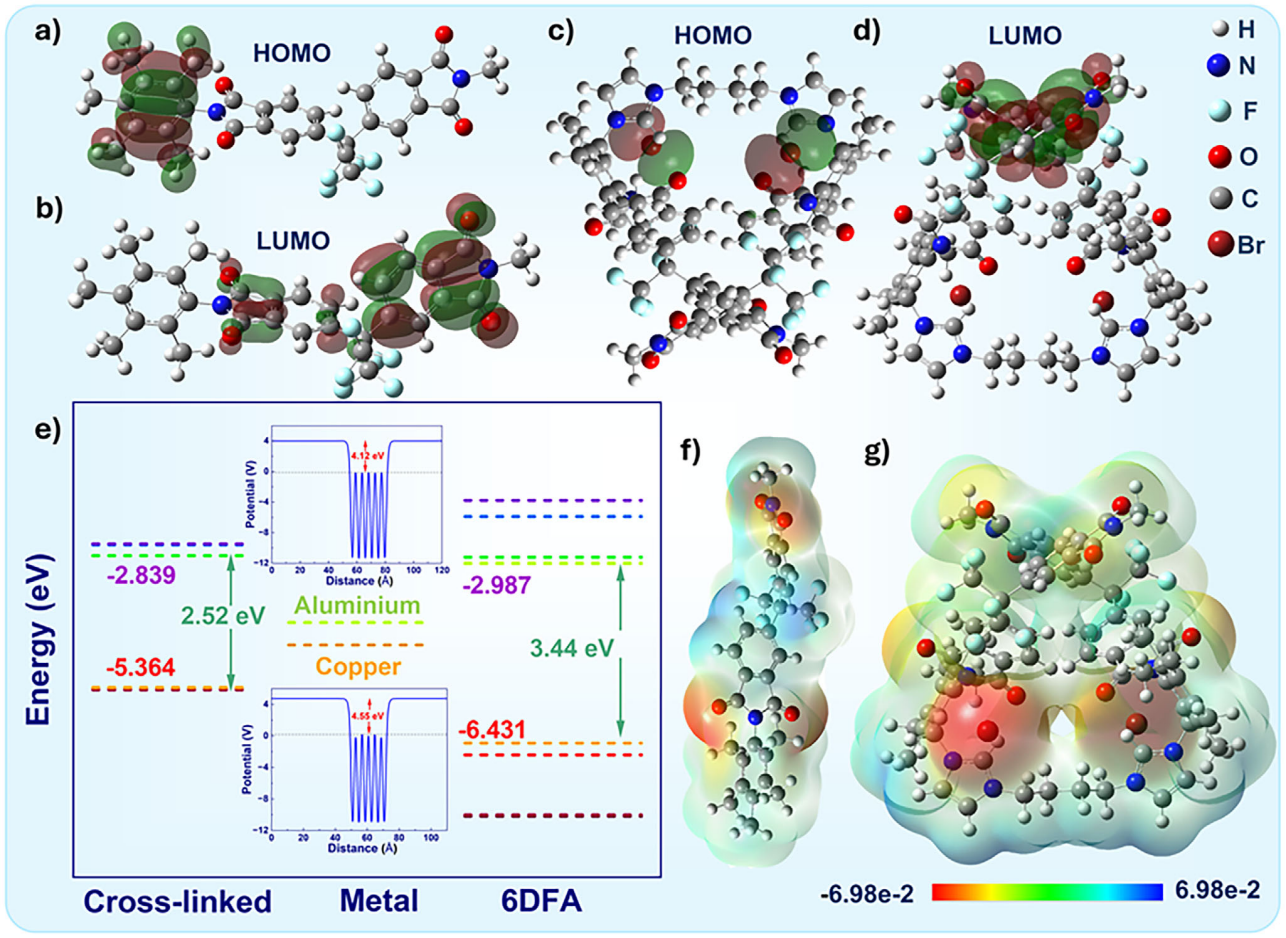
Spatial distributions of frontier orbitals for single‐chain models: (a, b) pristine 6FDA‐durene PI and (c, d) crosslinked Im‐6FDA‐durene PI (HOMO, LUMO). (e) Energy‐level diagram of HOMO and LUMO for both polymers, with the work functions of Al (111) and Cu (111) shown in the inset. (f, g) ESP maps of 6FDA and the cross‐linked polymer, respectively (blue positive, red negative).

Furthermore, DFT calculations were carried out to probe the electronic and structural properties of 6FDA and its cross‐linked polymer (Figure 3). The optimized geometries appear in Figure 3a–d, and the atomic coordinates and charges of the optimized cross‐linked polymer are listed in Table S1. Because amorphous polymers exhibit intrinsic structural disorder, precise quantification of charge transfer is difficult and typically limited to statistical estimates. To obtain clearer insight into electron‐donating or electron‐withdrawing tendencies, we evaluated the polymer's monomer unit (single chain segment) using established methods [46]. Additionally, given the insulating nature of PI‐like polymers, charge carriers remain localized along the polymer backbone [47]. Evaluating the monomer therefore provides a reasonable proxy for the electronic behavior of the amorphous system. Calculated bond angles for F─C─F and C─C─F were 107° and 111°, respectively, and the average C─F bond length was 1.34 Å. These values indicate covalent F─C interactions [48]. Harmonic frequency analyses (FTIR) for both polymer models showed no imaginary modes, confirming that all stationary points are true minima on the potential‐energy surface. Simulated FTIR results align with experiment (Figure 1d), and the corresponding peak assignments are summarized in Table S2.

We evaluate a molecule's charge‐transfer capability (donor/acceptor behavior) through its highest occupied molecular orbital (HOMO) and lowest unoccupied molecular orbital (LUMO) energies, which are critical indicators of electronic properties. Figure 3a–d show the spatial distributions and energies of the HOMO and LUMO for unmodified pristine 6FDA‐durene PI and the imidazolium‐mediated crosslinked 6FDA‐durene polymer, respectively. In general, nonbonding electrons occupy higher energy orbitals than bonding electrons and therefore have a greater propensity for donation [49]. The LUMO is broadly distributed along C─C and C═O frameworks for both pristine 6FDA‐durene PI (Figure 3a) and crosslinked Im‐6FDA‐durene PI (Figure 3c), informing its potential role in triboelectric response. For the HOMO, pristine 6FDA‐durene (Figure 3b) is mainly localized on the benzene ring, whereas in the crosslinked Im‐6FDA‐durene PI (Figure 3d), it is largely centered on Br‐associated sites. Electronegative atoms (N, O, F) withdraw electron density from neighboring atoms, creating localized electron‐rich regions and inducing negative dipoles; accordingly, the HOMO has substantial nonbonding character. Because nitrogen has a relatively low electron affinity, it tends to donate its lone‐pair electrons to a counter surface during contact‐separation, so N‐containing polymers typically display tribo‐positive behavior [50]. The lower HOMO energies observed in Figure S2 are consistent with this assignment: the nitrogen lone pairs reside primarily on the imidazole rings within the cross‐linked domains.

As shown in Figure 3e, the LUMO energy level changes only slightly after introducing the cross‐linked group, whereas the HOMO level shifts upward by ∼1 eV. According to *Koopmans' theorem*, the vertical ionization potential can be approximated by the negative of the HOMO energy; therefore, a higher (less negative) HOMO indicates easier electron removal from the neutral molecule [51]. In this framework, the cross‐linked polymer shows greater electron‐donating ability because of its higher HOMO energy. The LUMO of 6FDA lies slightly below that of the cross‐linked polymer, which indicates a greater tendency to accept electrons. Band gap is another key parameter that governs intramolecular charge‐transfer complex formation. As illustrated in Figure 3e, the band gap (Δ*E_g_* = *E_LUMO_*  − *E_HOMO_*) of the cross‐linked polymer is smaller than that of 6FDA [52]. Larger Δ*E_g_* values are typically associated with greater molecular stability and weaker intra‐/inter‐molecular charge‐transfer complex effects [53], and a wide band gap helps preserve localized electronic states, thereby stabilizing transferred electrons [54]. In this study, the band gaps of 6FDA and the cross‐linked polymer are found to be 3.44 and 2.52 eV, respectively, which remain sufficiently large to suppress unwanted electronic transitions.

Subsequently, the work functions of Al (111) and Cu (111) surfaces, used as secondary electrodes, are presented in Figure 3e (inside). The work function of a metal is defined as the energy difference between the Fermi level and the vacuum level, representing the minimum energy required for an electron to escape from the material surface. The calculated work functions are 4.12 eV for Al and 4.55 eV for Cu, which are consistent with previously reported values [54, 55]. Taking the vacuum energy as 0 eV, the Fermi level of Cu lies at −4.55 eV, which positions it above the HOMO but below the LUMO of 6FDA‐durene PI and crosslinked Im‐6FDA‐durene PI. The barrier height for electron injection at a metal‐polymer interface is determined by Equations (5) and (6), as shown previously in the energy‐level diagram in Figure 2d. The potential barrier heights were summarized for Cu (111) and Al(111) foils in Table S3 to assess the triboelectric charge density capability of each surface. For the crosslinked Im‐6FDA‐durene PI, the calculated barrier heights, $\varphi_{B}^{n}$, and $\varphi_{B}^{p}$ with Cu (111) foil were 1.71 and 0.814 eV, respectively. In contrast, the corresponding values for the 6FDA‐durene PI were 1.56 and 1.881 eV. These results identify the crosslinked Im‐6FDA‐durene PI as tribo‐positive in contact with Cu foil. For Al (111) foil, the calculated $\varphi_{B}^{n}$, and $\varphi_{B}^{p}$ values were 1.56 and 1.88 eV for the crosslinked Im‐6FDA‐durene PI, and 1.13 and 2.31 eV for the 6FDA‐durene PI. These results suggest that pristine 6FDA‐durene PI is a promising tribo‐negative material with Al. Driven by the energy level difference, electrons preferentially transfer from the Al foil to the LUMO of the 6FDA‐durene polymer molecules, whereas transfer occurs from the crosslinked Im‐6FDA‐durene polymer to the Cu foil. Moreover, the Cu foil exhibits a lower potential barrier at the interface with the crosslinked Im‐6FDA‐durene PI, thereby facilitating more efficient electron transfer compared to Al foil. These findings support selecting Cu foil as the tribo‐negative electrode.

Electrostatic potential (ESP) is a useful descriptor of the spatial variation in electrostatic potential over molecular surfaces, offering insight into charge transfer behavior. Also, by mapping regions of positive (blue) and negative (red) potential, ESP helps predict and rationalize intermolecular electrostatic interactions in TENG active materials. As shown in Figure 3f,g, ESP maps were computed for pristine 6FDA‐durene PI and the newly developed crosslinked Im‐6FDA‐durene PI, respectively. Electronegative atoms bearing lone pairs typically coincide with negative‐potential (red) regions. In our case, the H, N, and selected C sites in the imidazolium‐mediated crosslinked polymer predominantly appear in blue regions, indicating relatively positive electrostatic potential (Figure 3g). In contrast, the Br^−^ ions, C═O groups, and CF_3_ substituents of the crosslinked Im‐6FDA‐durene polymer appear in red, yellow, and green regions, respectively, indicating increasingly negative electrostatic potential [54]. The cross‐linking domains (e.g., imidazolium and aromatic rings) exhibit the most positive ESP, making them more prone to strong electrostatic interactions with Cu (111) during charge transfer.

Overall, our theoretical calculations confirm the tribo‐positive tendency of the crosslinked Im‐6FDA‐durene PI and provide fundamental insight into the polarity transition induced by the crosslinking process. The nitrogen‐containing imidazolium crosslinking domains generate localized electron‐rich regions within the polymer framework, thereby facilitating electron donation during contact electrification. Moreover, the HOMO distribution of the crosslinked Im‐6FDA‐durene PI is predominantly localized around the Br‐associated and imidazolium‐mediated crosslinking domains, whereas pristine 6FDA‐durene PI exhibits HOMO localization mainly on the benzene rings. This redistribution of the frontier orbital configuration shifts the HOMO level toward higher energies, facilitating electron removal from the polymer surface. Furthermore, the interfacial barrier‐height calculations reveal more favorable charge transfer from the crosslinked polymer to the Cu surface, further supporting its tribo‐positive behavior. In addition, the ESP maps exhibit positive electrostatic regions localized around the imidazolium‐linked crosslinking domains, promoting enhanced electrostatic interactions during contact electrification. Therefore, the combined HOMO/LUMO analysis, interfacial barrier‐height calculations, and ESP mapping consistently demonstrate that the crosslinked Im‐6FDA‐durene PI undergoes a distinct tribo‐polarity transition toward tribo‐positive behavior.

### 2.2 TENG Performance Evaluation

To enhance TENG performance, we synthesized a novel imidazolium‐mediated crosslinked variant of a 6FDA‐based polyimide and used it as the tribo‐positive layer in a two‐electrode device. While the unmodified polymer (pristine 6FDA‐durene PI) is inherently tribo‐negative, the newly designed imidazolium‐mediated crosslinking method was used to induce a reversal in surface polarity, effectively shifting its behavior to tribo‐positive. This transition is noteworthy, as there is a relative scarcity of tribo‐positive polymers compared to tribo‐negative ones. DFT results confirm the modified polymer's tribo‐donor character, establishing the crosslinked Im‐6FDA‐durene polymer as a new tribo‐positive candidate material. In evaluating TENG's potential as a green energy source, we prioritized adaptability to varying operating conditions, a factor that governs commercial viability. We measured performance under different mechanical loads and excitation frequencies, characterized capacitor charging–discharging across multiple capacitances, recorded *I–V* curves, and quantified power output versus resistive load. We also assessed durability at 50°C to represent hot‐city environments. Finally, we demonstrated practicality by lighting green LEDs and powering a small electronic device.

**FIGURE 4:**
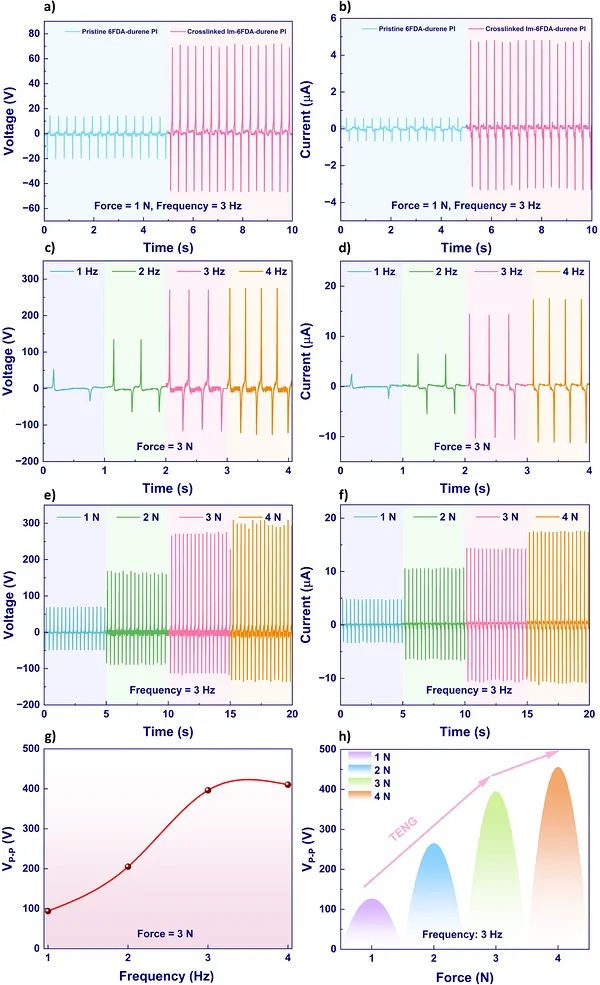
Electrical output performance of the triboelectric nanogenerator under different operating conditions. *V* and I outputs of (a) pristine 6FDA‐durene PI and (b) crosslinked Im‐6FDA‐durene PI at 3 Hz and an applied force of 3 N. Effect of operating frequency and applied force on the electrical output of the newly developed crosslinked Im‐6FDA‐durene PI. (c,d) *V* and I at 1–4 Hz under 3 N. (e,f) Corresponding outputs under 1–4 N at 3 Hz. (g) *V_P‐P_* as a function of frequency (3 N). (h) *V_P‐P_* as a function of force (3 Hz).

To elucidate the effect of chemical crosslinking on both triboelectric performance and polarity, pristine 6FDA‐durene PI and crosslinked Im‐6FDA‐durene PI were systematically evaluated under identical operating conditions under a force of 1 N and frequency of 3 Hz. As shown in Figure 4a, the pristine PI‐based device delivered a *V* of ∼20.1 V and an I of ∼ 0.6 µA. In contrast, the crosslinked Im‐6FDA‐durene PI exhibited a pronounced enhancement in electrical output, reaching ∼75.5 V and ∼4.7 µA, corresponding to a threefold increase in voltage and an order‐of‐magnitude improvement in current (Figure 4b). Beyond output enhancement, crosslinking induced a clear transition in triboelectric polarity. Under the same electrical connection configuration, the pristine 6FDA‐durene PI displayed dominant negative *V* peaks, characteristic of tribo‐negative behavior, whereas the crosslinked polymer showed dominant positive peaks, confirming its tribo‐positive nature. This experimental observation is independently supported by surface potential measurements (Figure S3), where the pristine 6FDA‐durene PI exhibits a negative surface potential of −0.23 kV, while the crosslinked Im‐6FDA‐durene PI shows a positive surface potential of +0.87 kV. Importantly, these experimental findings are in excellent agreement with our DFT calculations discussed earlier, which predict an upward shift in surface electrostatic potential and enhanced electron‐donating character upon imidazole‐based crosslinking (Figure 3). The consistent correlation between theoretical predictions, surface potential, and electrical output measurements provides compelling evidence that crosslinking effectively tailors both the triboelectric polarity and charge‐generation capability of the polymer.

The 50% bromination level of our polymer, used in this work, represents an optimized threshold; it successfully enables crosslinking on both sides of the polymeric backbone, yielding a structurally sound, well‐organized crosslinked network without compromising the macromolecular integrity. To incorporate a higher density of imidazolium groups, a higher degree of precursor bromination is required. Based on our previously reported works on polyimide modifications, an excessively high bromination load induces severe degradation of the polyimide backbone [41]. Furthermore, while increasing the density of imidazolium groups could theoretically be achieved by impregnating the matrix with “free” imidazolium‐based ionic liquids (ILs) as plasticizers, these composite materials suffer from severe long‐term stability issues due to the inevitable leaching of the unbonded IL under continuous cyclic deformation or harsh environmental conditions.

As a next step, we examined the effect of excitation frequency on the output voltage of the TENG under low‐frequency operation (1–4 Hz), while maintaining a constant applied force of 3 N. As shown in Figure 4c, increasing the frequency from 1 to 3 Hz resulted in a pronounced enhancement in the *V*. At 1 Hz, the peak voltage was approximately 59 V, rising to 140 V at 2 Hz and reaching a maximum of 280 V at 3 Hz, an overall increase of about 474%. According to Equation (1), *V* is governed by the instantaneous electrode separation, *x*(t), and the surface charge density, σ, and is not directly dependent on load frequency [56]. The observed frequency‐dependent enhancement is attributed to charge dynamics during successive contact‐separation cycles. At higher frequencies, the reduced interval between cycles minimizes charge leakage, enabling greater charge retention and accelerating the approach to electrostatic equilibrium, thereby increasing *V* [57]. However, when the frequency is increased to 4 Hz, the output maintains around 285 V, which can be attributed to the shortened contact‐separation interval that prevents complete neutralization of the charges accumulated on the film surface; thus, the achievable *V* is stable [58].

A similar frequency‐dependent trend was observed in the output I (Figure 4d). At 1 Hz, the current was ∼2.5 µA, increasing to 6.5 µA at 2 Hz and 14.4 µA at 3 Hz, representing a 580% increase relative to 1 Hz. At 4 Hz, the I reached ∼17.5 µA, again showing minimal decrease compared to the 3 Hz case. This increasing trend up to 4 Hz can be explained by Equation (2), where the I is directly related to the relative velocity of the contact‐separation motion *v*(*t*). Higher excitation frequencies produce faster contact and separation events, increasing the number of charge transfer cycles per unit time and reducing the opportunity for charge dissipation between cycles [59].

To investigate the effect of applied pressure on the tribo‐positive film, normal forces ranging from 1 N to 4 N were applied at a constant frequency of 3 Hz, while keeping all other experimental parameters unchanged (Figure 4e, f). Both *V* and I increased consistently with the applied force. As shown in Figure 4e, *V* exhibited a clear positive correlation with pressure, with measured values of approximately 75.5, 172.5, 280, and 317 V for forces of 1, 2, 3, and 4 N, respectively. Similarly, I ​ followed the same increasing trend (Figure 4f), rising from 4.7 µA at 1 N to 17.3 µA at 4 N, with intermediate values of 10.5 µA and 14.4 µA for 2 N and 3 N, respectively. The enhancement is attributed to elastic deformation of both the dielectric film and the counter electrode under higher loads, which increases the real contact area and promotes more effective charge transfer [60]. An increased contact area reduces micro‐ and nano‐scale gaps or defects (Figure S4a), enabling more efficient charge trapping and, consequently, enhancing triboelectric charge generation [61]. The underlying mechanism can be interpreted using the parallel‐plate capacitance model (Equation (4)), where increased force compresses the film, reducing the dielectric thickness (*d*) and allowing greater charge storage per cycle. Simultaneously, asperity flattening and improved surface conformity increase the effective contact area (*S*), further enhancing capacitance (*C*). This finding is in good alignment with other research [62]. Additionally, *V_P‐P_* was evaluated for both varying force and frequency. *V_P‐P_* increased with frequency, reached a maximum at 3 Hz under a 3 N load, and then stabilized (Figure 4g). Also, *V_P‐P_* showed a rising trend with rising force at 3 Hz (Figure 4h). Together, these results indicate a frequency‐dependent optimum and enhanced charge transfer at higher normal loads.

**FIGURE 5:**
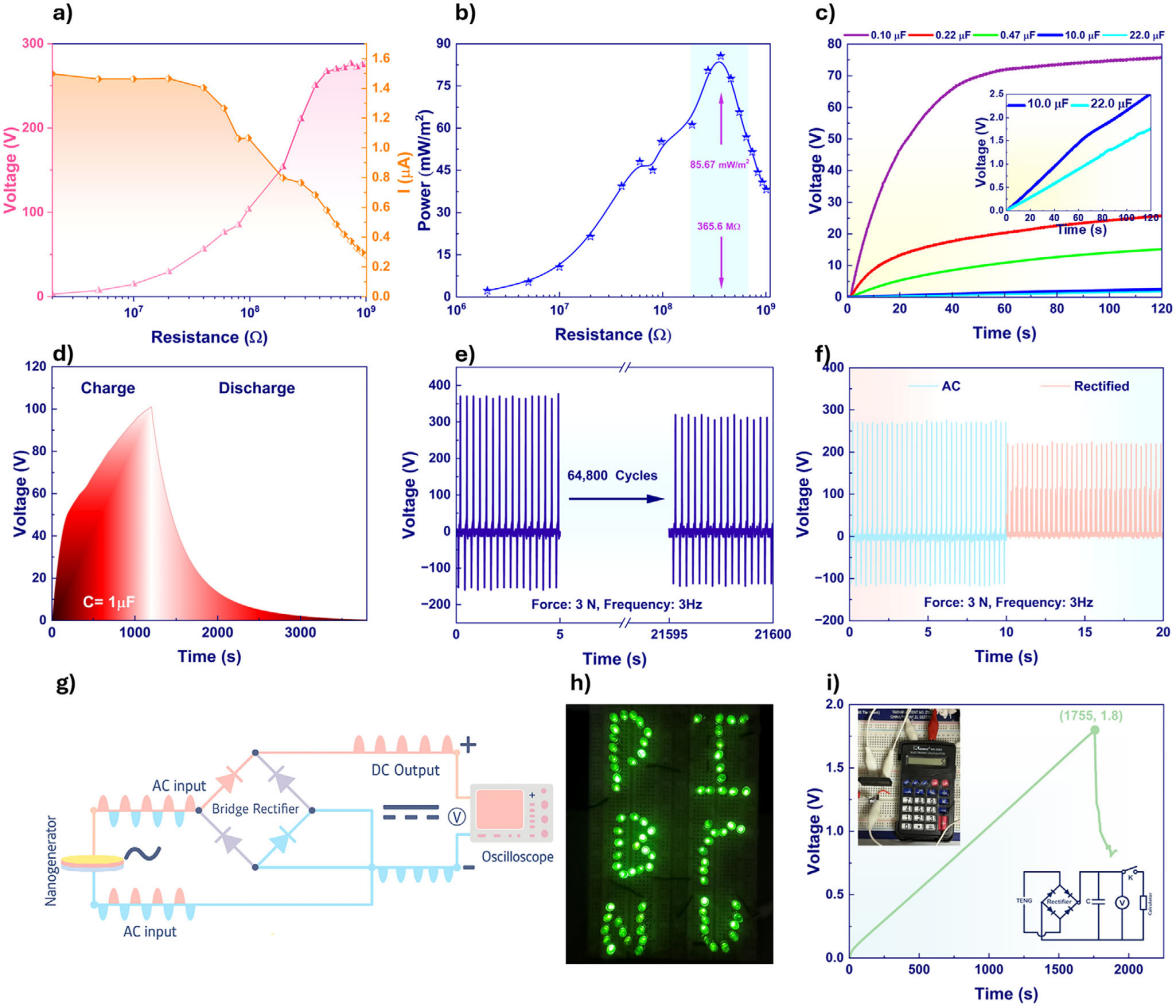
Output performance and demonstration of the crosslinked Im‐6FDA‐durene PI under 3 N at 3 Hz. (a) V and I versus load resistance. (b) Output power showing a peak of 85.67 mW/m^2^ at 365.6 MΩ. (c) Capacitor charging curves for different capacitances. (d) Charging‐discharging profile of a 1 µF capacitor measured under a 455 MΩ load resistance. (e) Durability over 64 800 cycles at 50°C. (f) *V_AC_* and rectified *V_DC_* under 3 N, 3 Hz. (g) Schematic of the bridge rectifier circuit used for AC‐to‐DC conversion and measurement. (h) Lighting of 81 green LEDs forming the “PI, Br, NU” pattern, and (i) powering a small electronic device such as a calculator with crosslinked Im‐6FDA‐durene PI with charge‐discharge graph.

In practical applications, the electrical output performance, particularly the power output, is a critical determinant of device functionality. To comprehensively evaluate crosslinked Im‐6FDA‐durene PI, we conducted systematic assessments of the output *V‐I* characteristics and power generation capability under varying load resistances (2 MΩ–10 GΩ), along with charging–discharging behavior of capacitors, LEDs illumination, and long‐term operational stability. Performance optimization was first investigated through impedance matching and peak power analysis of the crosslinked Im‐6FDA‐durene PI. Output measurements were performed at 3 Hz and 3 N under varying external load resistances (Figure 5a). At low resistance values, the output current remained nearly constant; however, with increasing resistance, it decreased from ∼1.47 µA at 2 MΩ to 0.28 µA at 10 GΩ. In contrast, the output voltage exhibited an opposite trend, rising from 3 V at 2 MΩ to 277.82 V at 10 GΩ. The corresponding output power initially increased with resistance, reached a maximum, and then declined. The highest instantaneous power density of 85.67 mW/m^2^ was obtained at an optimal load of 365.6 MΩ (Figure 5b). This behavior is consistent with the maximum power transfer theorem, which dictates that peak power occurs when the external load matches the device's internal impedance. The output power density of the crosslinked Im‐6FDA‐durene PI‐based TENG was systematically compared with recently reported tribo‐positive polymer‐based TENGs, including a previously reported tribo‐positive PI‐based system, as reported in Table S4. Based on the work of Zhen Pan et al. [63] on tribo‐positive PI‐based TENGs, structural modification was achieved by integrating electron‐donating amide groups directly into the molecular backbone, resulting in enhanced triboelectric performance. While their molecular backbone design represents an important milestone, our imidazolium‐crosslinked 6FDA‐Durene system offers distinct advantages in terms of preparation cost‐effectiveness and manufacturing scalability. Polyimides modified directly at the backbone level typically require complex, multi‐step monomer synthesis and precise stoichiometric control to achieve high molecular weights, followed by energy‐intensive, high‐temperature thermal imidization of unstable poly(amic acid) precursors directly on the substrate. In contrast, our preparation method completely bypasses complex backbone synthesis by utilizing a post‐functionalization approach on an already pre‐imidized and highly soluble base polymer. By simply introducing the bis‐imidazolium crosslinker into the 6FDA‐Durene solution, polarity reversal is achieved concurrently with low‐to‐moderate‐temperature film casting and straightforward solvent evaporation, significantly reducing raw material and operational energy costs. Consequently, this material is highly viable for large‐scale production because the formulation exists as a stable, single‐phase homogeneous casting solution processed under mild conditions and is fully compatible with high‐throughput industrial liquid‐phase manufacturing methods such as roll‐to‐roll web coating, slot‐die coating, and continuous doctor‐blade casting. This processing simplicity enables the continuous fabrication of robust, large‐area tribo‐positive films on flexible substrates using standard manufacturing infrastructure without requiring specialized high‐temperature curing lines.

The energy storage capability of the sample was evaluated by integrating it into a circuit comprising commercial capacitors and a bridge rectifier. The system operated under an impact force of 3 N at 3 Hz, with the rectified output applied to capacitors of 0.1, 0.22, 0.47, 10, and 22 µF. Each capacitor was charged for 120 s, achieving maximum voltages of 75.66, 25.78, 15.18, 2.52, and 1.76 V, respectively (Figure 5c). For clarity, the inset picture provides a zoomed view of the 10 and 22 µF results. Continually, as shown in Figure 5d, the rectified output was employed for repeated charging and discharging of a 1 µF capacitor over a duration of 3800 s, achieving a peak voltage of 100 V under an applied force of 3 N and a frequency of 3 Hz. To ensure controlled and measurable energy release, a high‐value resistor (455 MΩ) was connected in series with the capacitor during the discharge mode. Under this configuration, the stored energy was sustained over a discharge period of approximately 2625 s. These results demonstrate the effective energy storage capability of the crosslinked polymer‐based TENG, as well as its ability to retain and release harvested energy in a stable and regulated manner. Also, importantly, energy converters must deliver stable and continuous output over long operational periods and in harsh environments, requirements that are critical for efficient, reliable energy harvesting, particularly in high‐temperature settings. Prolonged frictional contact at elevated temperature in mechanical nanogenerators can alter surface morphology and reduce performance and lifespan [64].

The influence of relative humidity and temperature on the TENG performance was systematically investigated. For these purposes, a sealed chamber was designed to generate controlled humidity and temperature environments using a humidifier and a heater, as presented in Figure S5. During the humidity‐dependent measurements, the chamber temperature was maintained within the range of 28.9–31.1°C, while the relative humidity was varied from 37% to 95% (Figure S6). As the relative humidity increased from 37% to 95%, the voltage gradually decreased from 285 to 33 V. The reduction in electrical output can be mainly attributed to the adsorption of a small amount of water molecules on the polymer surface, leading to the formation of a conductive moisture layer that facilitates charge dissipation and suppresses effective triboelectric charge accumulation. In addition, the presence of absorbed moisture can partially screen surface charges and reduce the interfacial charge transfer efficiency during contact electrification. Nevertheless, even under an extremely high relative humidity of 95%, the device still generated an acceptable electrical output (Figure S6), demonstrating its stable operation under harsh humid conditions. This humidity tolerance is mainly associated with the hydrophobic nature of the sample, which originates from the presence of fluorinated C─F bonds, confirmed by the XPS and FTIR analyses. The hydrophobic surface effectively suppresses excessive moisture adsorption, thereby helping preserve the triboelectric performance even in highly humid environments [65].

Additionally, the temperature‐dependent performance of the crosslinked Im‐6FDA‐durene PI based TENG was systematically investigated over the range of 25–80°C. As shown in Figure S7, the voltage output increased continuously as the temperature increased from room temperature (25°C) to 70°C, showing an enhancement of approximately 52%. Notably, the most significant improvement was observed in the range of 50–70°C. However, when the temperature reached 80°C, the output decreased by approximately 8% compared with that at 70°C, indicating the onset of thermally induced charge dissipation (Figure S7). Although the highest electrical output was obtained at 70°C, prolonged operation at such elevated temperatures will adversely affect long term durability and operational stability. The reason for this performance trend can be explained by the fact that the electrical output initially increased with increasing temperature due to the reduction in surface humidity, which minimizes the adverse effect of moisture on the triboelectric performance. In addition, higher temperatures promote electron transfer by increasing the electron energy level and enhancing the probability of electrons escaping from the potential well, thereby generating more transferred charges during contact electrification and improving the electrical output. However, further temperature increase led to a gradual decline in performance due to charge dissipation induced by thermionic emission. In general, our results demonstrate that the crosslinked Im‐6FDA‐durene PI‐based TENG exhibits good stability and reliable performance under harsh environmental conditions [66].

In addition, we evaluated the durability of the crosslinked Im‐6FDA‐durene PI under 3 N and 3 Hz at 50°C. 50°C was selected as the durability testing condition because it provides a balance between high electrical performance and a realistic high‐temperature environment in the world. As shown in Figure 5e, the device retained 88% of its initial *V_p‐p_* after 64 800 cycles (6 h). Based on the SEM images obtained before and after the durability test, slight surface morphological changes were observed following cyclic operation. Prior to the durability test, the polymer surface already exhibited several micro/nano‐defects, which are attributed to the fabrication process of the polymer film (before testing, Figure S4a). During film preparation, trapped air bubbles and localized structural inhomogeneities generate micro/nano‐scale surface deformations. These surface features positively contribute to the triboelectric performance by increasing the effective contact roughness and interfacial contact area, which is consistent with the enhanced output observed under higher applied forces. However, after prolonged cyclic operation, some of these micro/nano‐deformed regions gradually evolved into localized abrasion and surface deformation areas, as highlighted in Figure S4b. These morphological changes are attributed to cumulative mechanical fatigue, repeated contact‐separation impacts, and localized stress concentration during continuous operation. In addition, long‐term cyclic loading accelerates slight surface aging of the polymer, further contributing to gradual interfacial degradation. As a result, the effective contact area and charge transfer efficiency were slightly reduced, leading to the observed 12% decay in triboelectric performance after extended cycling. Importantly, no severe delamination or catastrophic cracking was observed after the durability test, further confirming the good structural integrity and long‐term stability of the crosslinked Im‐6FDA‐durene PI‐based TENG.

This selection for 50°C was further supported by literature reports on environmental conditions in hot‐climate regions. Extremely high environmental temperatures are observed in many inhabited regions worldwide. Major cities such as Phoenix (USA), Delhi (India), and Kuwait City (Kuwait) frequently experience summer temperatures exceeding 40–45°C, with extreme events reaching 50–54°C. These observations demonstrate that temperatures approaching 50°C represent realistic environmental conditions for technologies intended for deployment in hot‐climate regions [67]. These results indicate good stability at 50°C and support the polymer's suitability in hot‐climate regions.

Figure 5f shows the cross‐linked TENG's native AC voltage (*V_AC_*) and its rectified output (*V_DC_*) under a 3 N force at 3 Hz; Figure 5g illustrates the circuit used to measure the *V_DC_*. In AC mode, periodic contact‐separation between the triboelectric layers induces alternating potentials across the electrodes; this drives bidirectional electron flow. For applications requiring a stable and unidirectional supply, such as charging capacitors, powering low‐voltage electronics, or integration with energy storage systems, the AC signal is converted to DC using a full‐wave bridge rectifier. The rectified *V_DC_* is inherently lower than the peak *V_AC_*, primarily due to voltage drops across the diodes in the rectification stage. In our measurements, this resulted in a reduction of approximately 20% in *V_DC_* compared to the *V_AC_*, as shown in Figure 5f. Furthermore, the device successfully powered 81 green LEDs under a low mechanical input of 3 N at 3 Hz (Figure 5h and Video S1). Additionally, the crosslinked Im‐6FDA‐durene PI‐based TENG demonstrated practical energy‐supplying capability by directly powering a commercial calculator under an operating force of 3 N and a frequency of 3 Hz. The harvested output was rectified and stored using a 440 µF capacitor, enabling stable device operation. The corresponding capacitor charging and discharging characteristics are presented in Figure 5i. The capacitor was charged to 1.8 V within 1755 s, followed by a discharge duration of approximately 123 s, confirming effective energy accumulation and release. A video demonstration of the calculator powered by the TENG is provided in Video S2.

## 3 Conclusion

The present study demonstrates the design and development of a polarity‐engineered, tribo‐positive polyimide via imidazolium‐mediated cross‐linking of typical 6FDA‐durene polyimide. We have synthesized selectively brominated 6FDA‐durene PI and successfully crosslinked it via a bisimidazole crosslinker having a flexible –(CH_2_)_4_‐ spacer chain, yielding a flexible, transparent, and free‐standing film of Im‐6FDA‐durene PI, which enabled excellent TENG measurements.  Interestingly, introduction of imidazolium moieties in the crosslinked matrix of 6FDA‐durene PI reverses its behavior from tribo‐negative pristine 6FDA‐durene PI to tribo‐positive, in agreement with DFT, which attributes the polarity reversal to electron donor imidazolium units. Under varying mechanical conditions, the electrical output rises with force; in the case of different frequencies, it shows the maximum at 4 Hz, where the device reaches *V*, 285 V and I, 17.5 𝜇A. At 3 N and 3 Hz, the device delivers a peak power density of 85.67 mW/m^2^ at an optimal 365.6 MΩ load, charges multiple capacitors, and powers 81 green LEDs and small electronics like a calculator. Furthermore, the output performance of the Im‐6FDA‐durene PI‐based TENG was systematically evaluated under harsh environmental conditions, including different temperatures and humidity levels, to assess its suitability for operation in hot‐humid environments. Continuously, durability tests retained 88% of *V_P‐P_* after 64 800 cycles at 50°C. Overall, the newly developed crosslinked Im‐6FDA‐durene PI, a tribo‐positive polymer, expands a limited tribo‐positive library and, with its thermal robustness, stands out as a strong candidate for triboelectric nanogenerators in hot climate operation. Moreover, owing to the superhydrophobic nature and chemical stability of the crosslinked Im‐6FDA‐durene PI surface, this polymer represents a promising candidate for future liquid‐solid triboelectric studies and related energy‐harvesting applications.

## 4 Experimental Section

### 4.1 Materials

4,4′‐(hexafluoroisopropylidene)diphthalic anhydride (6FDA) was purchased from Apollo Scientific (Stockport, UK), 2,3,5,6‐tetramethylbenzene‐1,4‐diamine (durene) was obtained from BLDpharm (Shanghai, China), and *N*‐bromosuccinimide (NBS) and 1,1,2,2‐tetrachloroethane were obtained from Sigma‐Aldrich (Darmstadt, Germany) and used without further purification. 6FDA and durene were dried under vacuum at 60°C for 24 h before polymerization. Imidazole, triethylamine, and acetic anhydride were purchased from Aldrich Chemical Co. Dimethylformamide (DMF, 99.8% anhydrous) was procured from Sigma‐Aldrich. All remaining chemicals were obtained from standard commercial suppliers and used as received.

### 4.2 Synthesis of 1,4‐di(1H‐Imidazol‐1‐yl)Butane(Bisimidazole)

Synthesis of 1,4‐di(1H‐imidazol‐1‐yl)butane(bisimidazole) was conducted according to our previously reported work [30]. 4 g of imidazole (58.75 mmol) was dissolved in THF (50 cm^3^) in a two‐necked flask with an N_2_ inlet, condenser, and a magnetic stirrer. Then, 4.2 g of NaH (176.26 mmol) was added to the solution at 0°C, and this mixture was stirred for 1 h at room temperature. 1,4‐Dibromobutane (3.52 cm^3^, 29.38 mmol) was slowly added with a syringe, and the reaction mixture was refluxed at 70°C overnight. The obtained mixture was cooled to room temperature, after which NaBr salt was removed by filtration and rinsed with THF. The filtrate was evaporated to dryness and extracted with methanol and hexane. The methanol layer was washed with hexane three times, and the product was isolated by rotary evaporation as white solid crystals.

### 4.3 Synthesis of 6FDA‐Durene Polyimide (PI)

6FDA–durene polyimide was synthesized through a typical polycondensation reaction, according to our previously reported work [68]. 8 g of 6FDA (18 mmol) and 2.96 g of durene (18 mmol) were dissolved in DMAc (60 cm^3^) using 250 cm^3^ two‐necked flasks equipped with a magnetic stirrer, N_2_ inlet, and condenser. The reaction mixture was stirred for 12 h at 5°C in an ice bath, producing the corresponding polyamic acid. 5.3 cm^3^ of triethylamine (37.8 mmol) and 3.6 cm^3^ of acetic anhydride (37.8 mmol) were added to the reaction mixture. Then this mixture was heated at 110°C for 3 h and stirred vigorously to achieve complete imidization of the polyamic acid, yielding the polyimide. The viscous mixture obtained after this step was cooled to room temperature, dissolved in 10 cm^3^ DMAc, and precipitated into methanol (400 cm^3^). 6FDA‐durene polymer was obtained as white polymer beads after filtration of the resultant mixture, several rinses with deionized water, and drying for 48 h under vacuum at 80°C.

### 4.4 Preparation of Brominated (Br‐PI)

A selective bromination of 50% of available benzylic positions at the durene moiety, which is two sites out of four sites, was conducted [41]. 6 g of 6FDA‐durene polyimide (10.5 mmol) and a catalytic amount of biphenyl peroxide (BPO) were dissolved in tetrachloroethane (30 cm^3^) in a 250 cm^3^ two‐necked flask with a magnetic stirrer, N_2_ inlet, and condenser. This mixture was heated at 85°C till complete dissolution; subsequently 4.11 g of N‐bromosuccinimide (23.1 mmol) was added to the reaction mixture and stirred for 12 h at the same temperature. The obtained red colored polymer solution was cooled to room temperature and precipitated into methanol (400 cm^3^). Then this solution was filtered, washed with deionized water several times, and dried for 48 h under vacuum at 80°C to give brominated polyimide (Br‐PI).

### 4.5 Preparation of Crosslinked IM‐6FDA‐Durene PI Membrane for TENG

The imidazolium‐mediated crosslinked 6FDA‐durene PI membrane was prepared by dissolving 0.2 g of Br‐PI in 2 mL of DMF by stirring the mixture at 500 rpm overnight at 40°C (Figure 1a). After the preparation of this dope solution, 0.06 g (1.1eq) of crosslinker 1,4‐di(1H‐imidazol‐1‐yl)butane(bisimidazole) was added to the solution at room temperature and stirred for another 15 min at 500 rpm until they were fully dissolved. The resultant solution was filtered through a cotton plug and cast on a glass plate using a doctor blade, as depicted in Figure 1a. After casting the membrane onto the glass, it was placed in an oven at 70°C for 24 h for solvent evaporation. Further, the membrane was left to dry under vacuum conditions at 90°C for 6 h. Once removed from the oven, the glass was allowed to cool for 30 min and then soaked in water to detach the ready membrane having a thickness of ∼50 µm.

### 4.6 DFT Calculation Details

DFT calculations were carried out using the Gaussian 16 W package [69]. Each polymer monomer chain was first subjected to full geometry optimization to ensure the attainment of minimum energy configurations. The optimizations were performed using the widely adopted B3LYP hybrid functional in combination with the 6–31++G(d,p) basis set. This level of theory has been extensively validated for polymer systems [70, 71]. To verify structural stability and the intrinsic feasibility of the adsorbents, vibrational frequency analyses were performed at the same theoretical level. All optimized structures were confirmed as true minima, exhibiting no imaginary frequencies [72]. Subsequently, single‐point calculations were performed to obtain surface electrostatic potentials and the HOMO and LUMO energies, providing insights into the electronic properties of the materials. In addition, natural bond orbital (NBO) analyses were performed to further elucidate the electronic structure and bonding interactions. The open‐source Quantum ESPRESSO [73] package was employed to calculate the work function of Al and Cu (111) foils using a 3 × 3 × 1 supercell. Since Paier et al. [74] demonstrated that the B3LYP functional performs poorly for metallic systems, the Perdew‐Burke‐Ernzerhof (PBE) exchange‐correlation functional was adopted instead. The convergence threshold for the total energy was set to 1.3 × 10^−^^6^ eV to ensure reliable accuracy.

### 4.7 Material Characterization and Testing

We examined our sample using several techniques to describe its structure and composition fully. The elemental composition of the sample surface was analyzed using XPS (NEXSA; Thermo Fisher Scientific, USA). An FT‐IR with a Nicolet iS10 FT‐IR spectrometer (Thermo Fisher Scientific Inc.) was used to study chemical bonds. A custom‐designed in‐house testing platform was employed to characterize the electrical performance of the Pristine 6FDA‐durene PI and crosslinked Im‐6FDA‐durene PI films used in the TENG device. The samples were cut into 40 mm × 70 mm substrates, maintaining an effective contact area of 40 × 50 mm, while the remaining portion served as a connection interface to the testing setup. The substrate was affixed to the platform using removable double‐sided tape to ensure consistent testing conditions across all samples. The platform featured a vertically oriented linear motor (LinMot DM01‐37×120F‐HP‐R‐195) operating at frequencies ranging from 1 to 4 Hz and applying contact forces between 1 and 4 N, enabling controlled contact–separation cycles. The *V* and I were recorded using a PicoScope 2000 series (Pico Technology Ltd) and a Stanford Research Systems SR570 low‐noise current preamplifier. To minimize ambient electromagnetic interference from light sources, Wi‐Fi, and communication networks, all tests were performed within a metallic Faraday cage. Capacitor charging‐discharging measurements were carried out using a Keithley 6514 electrometer. The same instrument was employed to evaluate the leakage current of the device, which was found to be approximately 8 nA, indicating low current loss and good electrical insulation of the fabricated structure. A Trek 542A electrostatic voltmeter was used to measure the surface potential of the samples.

## Conflicts of Interest

The authors declare no conflicts of interest.

## Supporting information




**Supporting File 1**: smll74486‐sup‐0001‐SuppMat.docx.

**Supporting File 2**: smll74486‐sup‐0002‐VideoS1.MOV.

**Supporting File 3**: smll74486‐sup‐0003‐VideoS2.mp4.

## Data Availability

The data that support the findings of this study are available from the corresponding author upon reasonable request.
