# Supplementary material for: Polarity‐Engineered, Tribo‐Positive Imidazolium‐Mediated Crosslinked 6FDA‐Durene Films for Triboelectric Nanogenerator
Source: Small. 2026 Jul 14;22(50):e74486. doi: 10.1002/smll.74486 (PMC13548995; doi:10.1002/smll.74486)
Supplement: Supplementary file 1 [file SMLL-22-e74486-s001.docx]

**Polarity-Engineered, Tribo-positive Imidazolium-mediated Crosslinked 6FDA-Durene Films for Triboelectric Nanogenerator**

*Sorour Faramarzi^1#^, Shabnam Yavari^1#^, Ukiliaiym Ospanova^2^, Adilet Shynggys^1^, Irshad Kammakakam^2*^, Haifeng Ji^3^, Jiayi Wang^3^, Zhen Wen^3^, Gulnur Kalimuldina^1*^*

^1^Department of Mechanical and Aerospace Engineering, School of Engineering and Digital Sciences, Nazarbayev University, Kabanbay Batyr Ave. 53, Astana 010000, Kazakhstan.

^2^Department of Chemistry, School of Sciences and Humanities, Nazarbayev University, 53 Kabanbay Batyr Ave., Astana, 010000, Kazakhstan

^3^Institute of Functional Nano and Soft Materials (FUNSOM), Joint International Research Laboratory of Carbon-Based Functional Materials and Devices, Soochow University, Suzhou 215123, China

^#^ As the first authors with the same authorship contribution.

* Corresponding authors: *gkalimuldina@nu.edu.kz,* [*irshad.kammakakam@nu.edu.kz*](mailto:irshad.kammakakam@nu.edu.kz)

**Table S1**. The atoms charge and coordination in cross-linked polymer structure after fully geometrical optimization by the B3LYP/6–311++G(d,p) computational level.

| *Symbol* | *charge* | *X(Å)* | *Y(Å)* | *Z (Å)* | *Symbol* | *charge* | *X(Å)* | *Y(Å)* | *Z (Å)* |
| --- | --- | --- | --- | --- | --- | --- | --- | --- | --- |
| *C* | *-0.016* | *-0.35198* | *2.623144* | *-0.11138* | ***C*** | *-0.172* | *0.604756* | *0.397704* | *6.054037* |
| *C* | *-0.023* | *-0.65655* | *3.677231* | *-0.96262* | ***C*** | *-0.39* | *0.179294* | *-1.04313* | *6.341084* |
| *C* | *-0.207* | *0.244284* | *4.145116* | *-1.90429* | ***H*** | *0.194* | *1.274263* | *0.75068* | *6.846886* |
| *C* | *-0.084* | *1.484821* | *3.499039* | *-2.00159* | ***H*** | *0.217* | *1.171822* | *0.43868* | *5.119316* |
| *C* | *-0.177* | *1.780303* | *2.43468* | *-1.13076* | ***N*** | *-0.379* | *1.269144* | *-2.01632* | *6.168457* |
| *C* | *-0.165* | *0.874393* | *1.985014* | *-0.17171* | ***H*** | *0.2* | *-0.17818* | *-1.14831* | *7.366749* |
| *C* | *0.823* | *-1.51555* | *2.388487* | *0.783687* | ***H*** | *0.252* | *-0.61829* | *-1.3725* | *5.665438* |
| *C* | *0.809* | *-2.02471* | *4.170831* | *-0.6395* | ***C*** | *0.325* | *1.769737* | *-2.33731* | *4.966636* |
| *C* | *0.236* | *-3.81263* | *3.374661* | *0.959497* | ***C*** | *-0.02* | *1.644166* | *-3.00022* | *7.069571* |
| *C* | *0.04* | *-4.11798* | *4.216598* | *2.040912* | ***N*** | *-0.381* | *2.538236* | *-3.42712* | *5.094986* |
| *C* | *0.088* | *-4.77683* | *2.541034* | *0.378882* | ***H*** | *0.263* | *1.658861* | *-1.75558* | *4.067574* |
| *C* | *-0.062* | *-5.42118* | *4.210045* | *2.564572* | ***C*** | *-0.02* | *2.432948* | *-3.87881* | *6.400913* |
| *C* | *-0.058* | *-6.07882* | *2.529352* | *0.917549* | ***H*** | *0.224* | *1.318309* | *-2.98928* | *8.094702* |
| *C* | *-0.011* | *-6.39033* | *3.343301* | *2.021524* | ***C*** | *-0.221* | *3.048085* | *-4.19838* | *3.937027* |
| *C* | *-0.408* | *-7.12998* | *1.621877* | *0.300328* | ***H*** | *0.225* | *2.922427* | *-4.77534* | *6.737414* |
| *C* | *-0.565* | *-7.20969* | *0.233824* | *0.963842* | ***C*** | *0.04* | *4.112168* | *-3.47814* | *3.135415* |
| *C* | *-0.1* | *2.592386* | *3.953223* | *-2.98532* | ***H*** | *0.26* | *2.167943* | *-4.43287* | *3.337527* |
| *C* | *1.067* | *2.035001* | *4.833252* | *-4.15482* | ***H*** | *0.216* | *3.426905* | *-5.13736* | *4.33777* |
| *C* | *1.066* | *3.591642* | *4.824842* | *-2.15036* | ***C*** | *0.236* | *3.807961* | *-2.97694* | *1.859672* |
| *C* | *-0.075* | *3.281157* | *2.738449* | *-3.65774* | ***C*** | *-0.062* | *5.415154* | *-3.32418* | *3.636563* |
| *C* | *-0.189* | *2.457601* | *1.710706* | *-4.14875* | ***C*** | *0.088* | *4.772825* | *-2.34215* | *1.067315* |
| *C* | *-0.124* | *4.669564* | *2.65646* | *-3.84864* | ***N*** | *-0.655* | *2.471871* | *-3.10836* | *1.346143* |
| *C* | *-0.102* | *2.97532* | *0.600108* | *-4.81727* | ***C*** | *-0.012* | *6.385228* | *-2.64771* | *2.870581* |
| *C* | *-0.113* | *5.171718* | *1.543573* | *-4.50137* | ***C*** | *-0.579* | *5.802844* | *-3.86785* | *4.995048* |
| *C* | *-0.121* | *4.347463* | *0.531975* | *-4.98008* | ***C*** | *-0.058* | *6.074747* | *-2.17962* | *1.581043* |
| *C* | *0.688* | *6.587049* | *1.1908* | *-4.82996* | ***C*** | *-0.627* | *4.435701* | *-1.85424* | *-0.32419* |
| *C* | *0.689* | *5.204179* | *-0.50325* | *-5.63433* | ***C*** | *0.823* | *1.51161* | *-2.07979* | *1.412216* |
| *C* | *-0.626* | *-4.43925* | *1.679235* | *-0.81749* | ***C*** | *0.817* | *2.021574* | *-4.18969* | *0.546407* |
| *H* | *0.24* | *-0.02681* | *4.987941* | *-2.52289* | ***C*** | *-0.594* | *7.76678* | *-2.45251* | *3.462326* |
| *H* | *0.218* | *2.747157* | *1.953405* | *-1.19369* | ***H*** | *0.215* | *6.799703* | *-4.31131* | *4.973638* |
| *H* | *0.24* | *1.130542* | *1.186602* | *0.514626* | ***H*** | *0.215* | *5.818733* | *-3.07861* | *5.755449* |
| *H* | *0.205* | *-8.10934* | *2.102501* | *0.346735* | ***H*** | *0.198* | *5.120499* | *-4.63933* | *5.346901* |
| *H* | *0.208* | *-6.92245* | *1.494903* | *-0.76326* | ***C*** | *-0.408* | *7.127081* | *-1.48575* | *0.732133* |
| *H* | *0.203* | *-7.96316* | *-0.38775* | *0.472641* | ***H*** | *0.219* | *4.932064* | *-2.46807* | *-1.08273* |
| *H* | *0.199* | *-6.25251* | *-0.29039* | *0.908167* | ***H*** | *0.205* | *3.368252* | *-1.89452* | *-0.52665* |
| *H* | *0.196* | *-7.47306* | *0.31112* | *2.02173* | ***H*** | *0.212* | *4.758514* | *-0.82247* | *-0.47458* |
| *H* | *0.225* | *1.387528* | *1.779409* | *-4.00581* | ***C*** | *-0.017* | *0.348805* | *-2.55596* | *0.617589* |
| *H* | *0.239* | *5.350236* | *3.424721* | *-3.51241* | ***O*** | *-0.634* | *1.670955* | *-1.0219* | *1.982638* |
| *H* | *0.225* | *2.325* | *-0.17958* | *-5.19467* | ***C*** | *-0.049* | *0.654227* | *-3.80609* | *0.09573* |
| *H* | *0.219* | *-4.94265* | *2.047739* | *-1.71705* | ***O*** | *-0.589* | *2.657704* | *-5.18573* | *0.307086* |
| *H* | *0.205* | *-3.37273* | *1.667865* | *-1.02824* | ***H*** | *0.212* | *8.348854* | *-1.70752* | *2.926528* |
| *H* | *0.212* | *-4.7541* | *0.645069* | *-0.66594* | ***H*** | *0.211* | *7.707409* | *-2.12651* | *4.503115* |
| *N* | *-0.656* | *-2.47628* | *3.356286* | *0.430258* | ***H*** | *0.213* | *8.341395* | *-3.38621* | *3.452313* |
| *N* | *-0.516* | *6.519613* | *-0.03957* | *-5.50424* | ***C*** | *-0.565* | *7.20911* | *0.033282* | *0.975101* |
| *F* | *-0.332* | *2.951859* | *5.751035* | *-1.43035* | ***H*** | *0.205* | *8.10568* | *-1.93508* | *0.913051* |
| *F* | *-0.337* | *4.289008* | *4.053166* | *-1.29201* | ***H*** | *0.208* | *6.919558* | *-1.66529* | *-0.32386* |
| *F* | *-0.353* | *4.492358* | *5.47192* | *-2.92244* | ***C*** | *-0.155* | *-0.87699* | *-1.96011* | *0.37852* |
| *F* | *-0.332* | *1.61246* | *6.045353* | *-3.75121* | ***C*** | *-0.092* | *-0.24474* | *-4.51817* | *-0.68014* |
| *F* | *-0.344* | *2.965245* | *5.035551* | *-5.10326* | ***H*** | *0.203* | *7.963442* | *0.489075* | *0.328125* |
| *F* | *-0.34* | *0.989672* | *4.224394* | *-4.75397* | ***H*** | *0.199* | *6.252694* | *0.521671* | *0.772931* |
| *O* | *-0.548* | *7.599898* | *1.801176* | *-4.5827* | ***H*** | *0.196* | *7.472532* | *0.258565* | *2.011612* |
| *O* | *-0.552* | *4.877426* | *-1.53561* | *-6.17164* | ***C*** | *-0.179* | *-1.78095* | *-2.65933* | *-0.41934* |
| *O* | *-0.586* | *-2.66049* | *5.059821* | *-1.14894* | ***H*** | *0.24* | *-1.13399* | *-1.00178* | *0.813964* |
| *O* | *-0.633* | *-1.67506* | *1.533328* | *1.628196* | ***C*** | *-0.123* | *-1.4841* | *-3.9244* | *-0.95793* |
| *C* | *-0.596* | *-5.81035* | *5.116413* | *3.712807* | ***H*** | *0.228* | *0.027208* | *-5.50022* | *-1.0381* |
| *H* | *0.215* | *-6.80625* | *5.537125* | *3.564088* | ***H*** | *0.218* | *-2.74702* | *-2.21435* | *-0.6171* |
| *H* | *0.215* | *-5.8297* | *4.57466* | *4.665426* | ***C*** | *-0.104* | *-2.58884* | *-4.63405* | *-1.78043* |
| *H* | *0.198* | *-5.12709* | *5.954892* | *3.833786* | ***C*** | *1.066* | *-2.02849* | *-5.8044* | *-2.65743* |
| *C* | *-0.221* | *-3.05455* | *5.134369* | *2.606965* | ***C*** | *1.067* | *-3.59185* | *-5.23922* | *-0.73973* |
| *H* | *0.26* | *-2.17382* | *5.189398* | *1.966504* | ***C*** | *-0.075* | *-3.27398* | *-3.65379* | *-2.76632* |
| *H* | *0.217* | *-3.43297* | *6.1485* | *2.725558* | ***F*** | *-0.332* | *-1.60886* | *-6.85676* | *-1.93142* |
| *N* | *-0.6381* | *-2.54621* | *4.721529* | *3.936142* | ***F*** | *-0.344* | *-2.95574* | *-6.26212* | *-3.51563* |
| *C* | *0.325* | *-1.78031* | *3.638195* | *4.121284* | ***F*** | *-0.34* | *-0.98041* | *-5.38618* | *-3.39835* |
| *C* | *-0.02* | *-2.4399* | *5.523272* | *5.061503* | ***F*** | *-0.331* | *-2.95555* | *-5.9294* | *0.211291* |
| *H* | *0.263* | *-1.67102* | *2.826154* | *3.423147* | ***F*** | *-0.352* | *-4.29087* | *-4.25929* | *-0.13162* |
| *C* | *-0.02* | *-1.65295* | *4.867456* | *5.951444* | ***F*** | *-0.353* | *-4.491* | *-6.07469* | *-1.30493* |
| *H* | *0.225* | *-2.92731* | *6.479417* | *5.131002* | ***C*** | *-0.19* | *-2.4477* | *-2.80262* | *-3.52023* |
| *H* | *0.225* | *-1.327* | *5.145752* | *6.938106* | ***C*** | *-0.124* | *-4.66162* | *-3.62801* | *-2.97752* |
| *N* | *-0.379* | *-1.28039* | *3.668273* | *5.365061* | ***C*** | *-0.102* | *-2.96199* | *-1.92087* | *-4.47228* |
| *Br* | *-0.845* | *0.629697* | *4.550186* | *2.68239* | ***H*** | *0.225* | *-1.37819* | *-2.82896* | *-3.36007* |
| *C* | *-0.172* | *-0.19202* | *2.78141* | *5.805205* | ***C*** | *-0.113* | *-5.16039* | *-2.73972* | *-3.91499* |
| *C* | *-0.39* | *-0.61688* | *1.316807* | *5.922492* | ***H*** | *0.239* | *-5.34419* | *-4.27291* | *-2.44386* |
| *H* | *0.2* | *0.159326* | *3.164852* | *6.764568* | ***C*** | *-0.121* | *-4.3335* | *-1.90046* | *-4.65243* |
| *H* | *0.252* | *0.609663* | *2.912392* | *5.069421* | ***H*** | *0.225* | *-2.30955* | *-1.27634* | *-5.04862* |
| *H* | *0.195* | *-1.29058* | *1.194253* | *6.778368* | ***C*** | *0.694* | *-6.57425* | *-2.49179* | *-4.33348* |
| *H* | *0.217* | *-1.17904* | *1.020305* | *5.032024* | ***C*** | *0.683* | *-5.18697* | *-1.08706* | *-5.57094* |
| *C* | *0.212* | *-7.77207* | *3.322646* | *2.643852* | ***N*** | *-0.516* | *-6.50331* | *-1.49646* | *-5.32202* |
| *H* | *0.211* | *-8.35167* | *2.453534* | *2.344448* | ***O*** | *-0.554* | *-7.58851* | *-3.0098* | *-3.93035* |
| *H* | *0.213* | *-7.71319* | *3.309575* | *3.734406* | ***O*** | *-0.546* | *-4.85739* | *-0.24409* | *-6.37219* |
| *H* | *0.208* | *-8.34893* | *4.212581* | *2.36569* | ***C*** | *-0.365* | *-7.65907* | *-0.95537* | *-6.01956* |
| *C* | *-0.365* | *7.677847* | *-0.75154* | *-6.02068* | ***H*** | *0.208* | *-7.58991* | *-1.16379* | *-7.08877* |
| *H* | *0.208* | *7.612066* | *-0.84818* | *-7.10592* | ***H*** | *0.226* | *-8.54701* | *-1.43248* | *-5.60792* |
| *H* | *0.226* | *8.564251* | *-0.17777* | *-5.75497* | ***H*** | *0.208* | *-7.71649* | *0.124778* | *-5.87526* |
| *H* | *0.208* | *7.735272* | *-1.74912* | *-5.58206* | ***Br*** | *-0.845* | *-0.63673* | *-3.62263* | *3.84229* |

**Table S2**. DFT calculated FTIR peaks.

| *related bonds streatching* | *Frequency (cm-1)* |
| --- | --- |
| *Out of plane cross-linked* | *609, 619* |
| *C-H out of plane* | *713, 720* |
| *C-N heterocyclic ring* | *1150* |
| *C-F* | *1184, 1187, 1108* |
| *C=C* | *1209,* |
| *C-N* | *1374, 1396* |
| *C=O* | *1750-1825* |
| *C-H* | *3016-3104* |

**Table S3**. The electron affinity, ionization energy, metal work function, and band gap of the polymer chain and electron and hole carrier injection barrier heights ($\varphi_{B}^{n}$, and $\varphi_{B}^{p}$) for Cu/Al-polymer interfaces.

| *Polymer* | $\boldsymbol{A (eV)}$ | $\boldsymbol{I (eV)}$ | $\boldsymbol{E}_{\boldsymbol{g}}\boldsymbol{(eV)}$ | $\boldsymbol{\varphi}_{\boldsymbol{M}}\boldsymbol{(eV)}$ | $\boldsymbol{\varphi}_{\boldsymbol{B}}^{\boldsymbol{p}}\boldsymbol{(eV)}$ | $\boldsymbol{\varphi}_{\boldsymbol{B}}^{\boldsymbol{n}}\boldsymbol{(eV)}$ |
| --- | --- | --- | --- | --- | --- | --- |
| *Cu (111)* | *-* | *-* | *-* | *4.55* | *-* | *-* |
| *6DFA* | *-2.987* | *-6.431* | *3.444* | *-* | *1.88* | *1.56* |
| *Cross-linked* | *-2.839* | *-5.364* | *2.524* | *-* | *0.814* | *1.71* |
| *Al (111)* | *-* | *-* | *-* | *4.12* | *-* | *-* |
| *6DFA* | *-2.987* | *-6.431* | *3.444* | *-* | *2.31* | *1.13* |
| *Cross-linked* | *-2.839* | *-5.364* | *2.524* | *-* | *1.24* | *1.28* |

**Table S4:** Comparison of Crosslinked Im-6FDA-durene PI with other tribo-positive materials.

| *Number* | *Material* | | *Voltage* | *Power density* | *Stability (cycles)* | *References* |
| --- | --- | --- | --- | --- | --- | --- |
|  | *Tribo-positive* | *Tribo-negative* |  |  |  |  |
| *1* | *PLA* | *Reed film* | *368 V* | *0.256 W/m^2^* | *10000* | [1] |
| *2* | *PEO /Ag@SiO2* | *Eco-flex* | *95 V* | *1.5 W/m2* | *10000* | [2] |
| *3* | *TPU/mica* | *PVDF/MXene* | *218 mV* | *1458 m W/m^2^* | *10000* | [3] |
| *4* | *Porous mica-Ecoflex* | *PI* | *68 V* | *55 m W/m^2^* | *720* | [4] |
| *5* | *PAN@ZIF-8* | *PTFE* | *260 V* | *1.91 W/m^2^* | *12000* | [5] |
| *6* | *Tribopositive elastic yarn* | *Silicone rubber tube* | *137 V* | *2.25 m W/m^2^* | *10800* | [6] |
| *7* | *PU* | *FEP* | *120 V* | *2.1 W/m^2^* | *20000* | [7] |
| *8* | *WPU@LiTFSI* | *Eco-flex* | *260 V* | *1.675 W/m^2^* | *50000* | [8] |
| *9* | *PU@g-C_3_N_4_* | *Eco-flex* | *465 V* | *2.89*  *W/m^2^* | *50000* | [9] |
| *10* | *PEI-treated cotton* | *PTFE* | *103 V* | *1.6 W/m^2^* | *4000* | [10] |
| *11* | *[SA/PEI] cotton fabric* | *Polyester fabric*  *[SA/PEI] cotton fabric* | *97.3 V* | *338 mW/m^2^* | *4000* | [11] |
| *12* | *PEI tribopositive yarn TENG* | *PDMS* | *45.6 V* | *5.518 mW/m^2^* | *4000* | [12] |
| *13* | *R2R Nylon-11* | *PET* | *306.2 V* | *3.24 W/m^2^* | *750* | [13] |
| *14* | *PI-d (BPADA-MMDABA)* | *fluorinated polyimide (FPI)* | *242 V* | *0.17 mW* | *2000* | [14] |
| *15* | *Crosslinked Im-6FDA-durene PI* | *Cu foil* | *280 V* | *85.67 mW/m^2^* | *64800* | *This work* |

**Figure S1**. The TGA profiles of pristine 6FDA-Durene (black) and crosslinked 6FDA-Durene (red).

**Figure S2**. The third and fourth HOMO levels of energy.

**Figure S3**. Measured surface potential of (a) pristine 6FDA-durene PI and (b) crosslinked Im-6FDA-durene PI.

**Figure S4.** SEM images of the crosslinked Im-6FDA-durene PI surface before (a) and after (b) the durability test. The green circles highlight the formation of localized micro/nano-defects, while the orange rectangles indicate regions of abrasion and surface deformation induced by repeated contact-separation cycles during long-term operation.

**Figure S5.** Photographs of the isolated environmental chambers designed for controlled temperature- and humidity-dependent measurements of the crosslinked Im-6FDA-durene PI based TENG. The temperature-controlled chamber was equipped with a heating system and a temperature sensor (a), while the humidity-controlled chamber was integrated with a humidifier system and a humidity sensor (b) to provide stable environmental conditions and continuous monitoring during the electrical output characterization.

**Figure S6**. Photographs showing the relative humidity variation from 37% to 95%, monitored using a humidity sensor (a). Output voltage generated by the TENG as a function of relative humidity under an applied force of 3 N and an operating frequency of 3 Hz (b).

**Figure S7.** The Voltage variation of crosslinked Im-6FDA-durene PI based TENG at different temperatures under an applied force of 3 N and an operating frequency of 3 Hz.

**References**

1.	Jian, G., et al., *Biodegradable triboelectric nanogenerator as a implantable power source for embedded medicine devices.* NPG Asia Materials, 2024. **16**(1): p. 12.

2.	Liu, X., et al., *Improvement in the output performance of polyethylene oxide-based triboelectric nanogenerators by introducing core–shell Ag@ SiO 2 particles.* Journal of Materials Chemistry C, 2022. **10**(1): p. 265-273.

3.	Li, W., et al., *High-performance triboelectric nanogenerators based on TPU/mica nanofiber with enhanced tribo-positivity.* Nano Energy, 2023. **114**: p. 108629.

4.	Wang, X., et al., *Mica's homo-positive-charging behavior enabled porous elastomer TENG for energy harvesting in high humidity.* Nano Energy, 2024. **119**: p. 109056.

5.	Pandey, P., et al., *Metal-organic frameworks-based triboelectric nanogenerator powered visible light communication system for wireless human-machine interactions.* Chemical Engineering Journal, 2023. **452**: p. 139209.

6.	Bai, Z., et al., *Constructing highly tribopositive elastic yarn through interfacial design and assembly for efficient energy harvesting and human-interactive sensing.* Nano Energy, 2022. **94**: p. 106956.

7.	Cheng, B.-X., et al., *High toughness, multi-dynamic self-healing polyurethane for outstanding energy harvesting and sensing.* ACS Applied Materials & Interfaces, 2023. **15**(50): p. 58806-58814.

8.	Bo, X., et al., *Surfactant Self‐Assembly Enhances Tribopositivity of Stretchable Ionic Conductors for Wearable Energy Harvesting and Motion Sensing.* Advanced Materials, 2024. **36**(32): p. 2403905.

9.	Islam, M.R., et al., *Amino-rich graphitic carbon nitride functionalized polyurethane nanomesh as highly tribopositive material for high-performance triboelectric nanogenerators and self-powered respiration monitoring wearables.* Chemical Engineering Journal, 2025. **518**: p. 164564.

10.	Bairagi, S., et al., *Polyethyleneimine (PEI)-treated multifunctional textile triboelectric nanogenerator: A scalable and cost-effective solution for self-powered electronics, energy harvesting and physiological movement monitoring.* Nano Energy, 2025. **139**: p. 110990.

11.	Das, S., A. Chowdhury, and S.W. Ali, *Wearable, machine washable, breathable polyethylenimine/sodium alginate layer-by-layer-coated cotton-based multifunctional triboelectric nanogenerators.* ACS Applied Materials & Interfaces, 2024. **16**(24): p. 31098-31113.

12.	Akram, W., et al., *A hierarchical core-shell tribopositive yarn TENG with electrospun polyethyleneimine (PEI) nanofibers for sustainable energy harvesting and human machine interface application.* Materials Today Energy, 2025: p. 102162.

13.	Fang, C., et al., *Highly tribo-positive Nylon-11 film fabricated by multiscale structural regulation through a roll-to-roll processing.* ACS Applied Materials & Interfaces, 2024. **16**(22): p. 29257-29266.

14.	Pan, Z., et al., *Preparation and Characterization of Amide-Containing Polyimide Films with Enhanced Tribopositivity for Triboelectric Nanogenerators to Harvest Energy at Elevated Temperatures.* Nanoenergy Advances, 2024. **4**(3): p. 284-299.
